# Supplementary material for: Spatiotemporal distributions of under-five mortality in Ethiopia between 2000 and 2019
Source: PLOS Glob Public Health. 2023 Mar 27;3(3):e0001504. doi: 10.1371/journal.pgph.0001504 (PMC10042344; doi:10.1371/journal.pgph.0001504)
Supplement: S1 Table — (DOCX) [file pgph.0001504.s001.docx]

**S1 Table:** Watanabe-Akaike information criterion (WAIC) values corresponding to different model specifications in all the five EDHS (2000-2019).

| Model specifications | WAIC | | | | |  |
| --- | --- | --- | --- | --- | --- | --- |
|  | **2000** | **2005** | **2011** | **2016** | **2019** | **2000-2019** |
| Access to health care facilities | 1958.282 | 1587.931 | 1644.531 | 1481.365 | 823.0869 | 7841.142 |
| Access to health care facilities + Population density | 1957.867 | 1584.087 | 1639.402 | 1483.952 | 825.3973 | 7835.37 |
| Access to health care facilities + Population density + Temperature | 1957.447 | 1580.261 | 1637.769 | 1483.811 | 826.7877 | 7836.536 |
| Access to health care facilities + Population density + Temperature + Precipitation | 1958.491 | 1581.455 | 1636.311 | 1483.975 | 827.8086 | 7837.635 |
| Access to health care facilities + Population density + Temperature + Precipitation + Access to cities | 1959.032 | 1583.185 | 1638.176 | 1483.565 | 829.6647 | 7838.83 |
| Access to health care facilities + Population density + Temperature + Precipitation + Access to cities + Distance to water body | 1961.393 | 1585.772 | 1638.914 | 1485.644 | 823.5629 | 7841.238 |
