# Supplementary figures and images for: Spatiotemporal distributions of under-five mortality in Ethiopia between 2000 and 2019
Source: PLOS Glob Public Health. 2023 Mar 27;3(3):e0001504. doi: 10.1371/journal.pgph.0001504 (PMC10042344; doi:10.1371/journal.pgph.0001504)

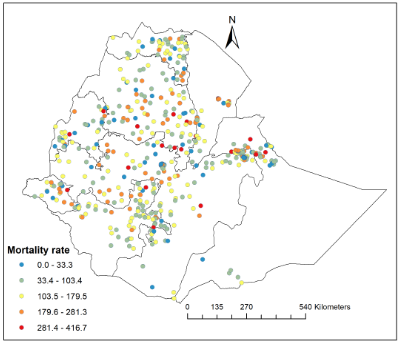
 A) **
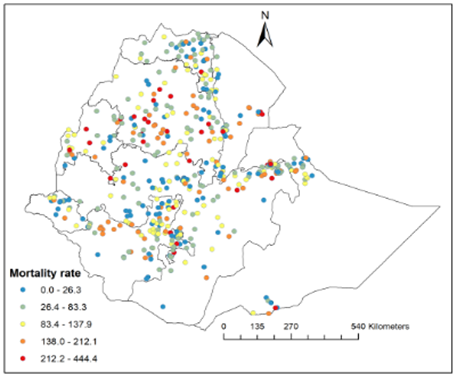
**B)


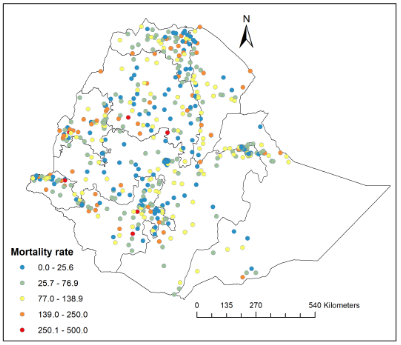
C)
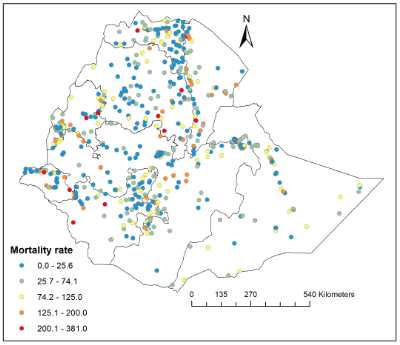
D)


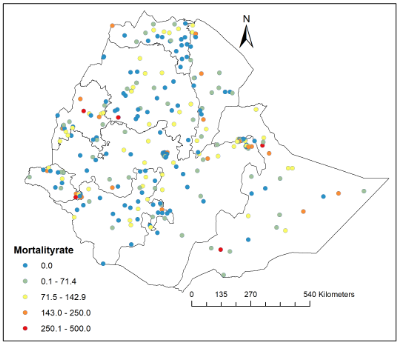
**E)**

*S1 Fig: Geographical locations of data points and under-five mortality in Ethiopia:* *2000(A), 2005(B), 2011(C), 2016(D), 2019(E).*

Supplement: S1 Fig — (DOCX) [file pgph.0001504.s002.docx]

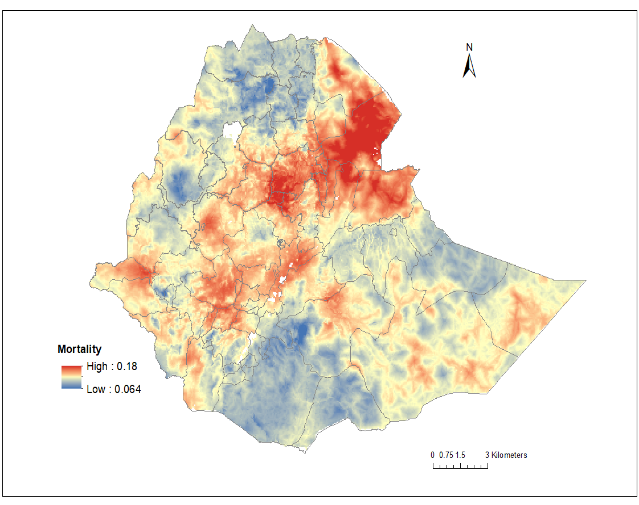
A)
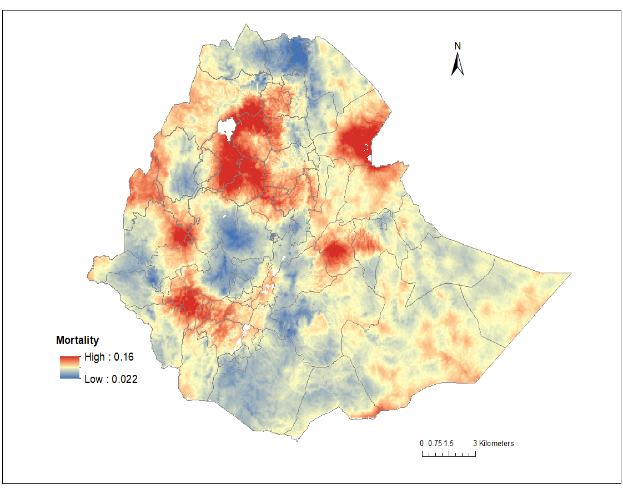
B)


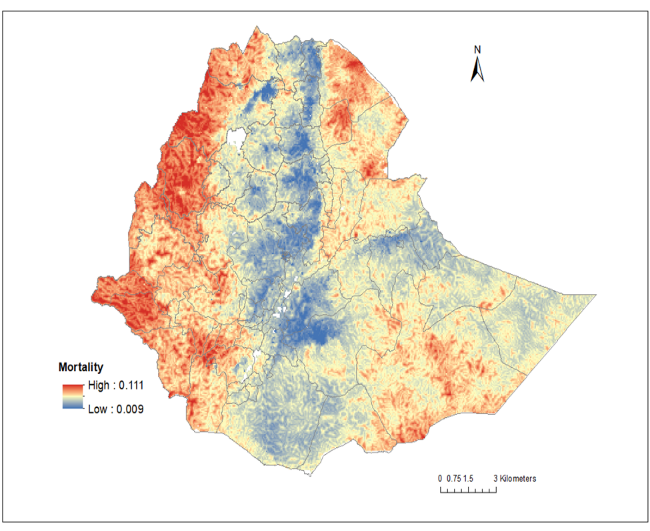
C)
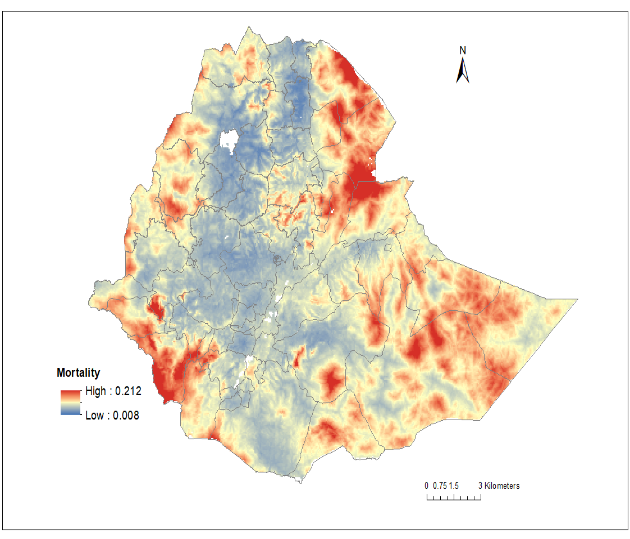
D)


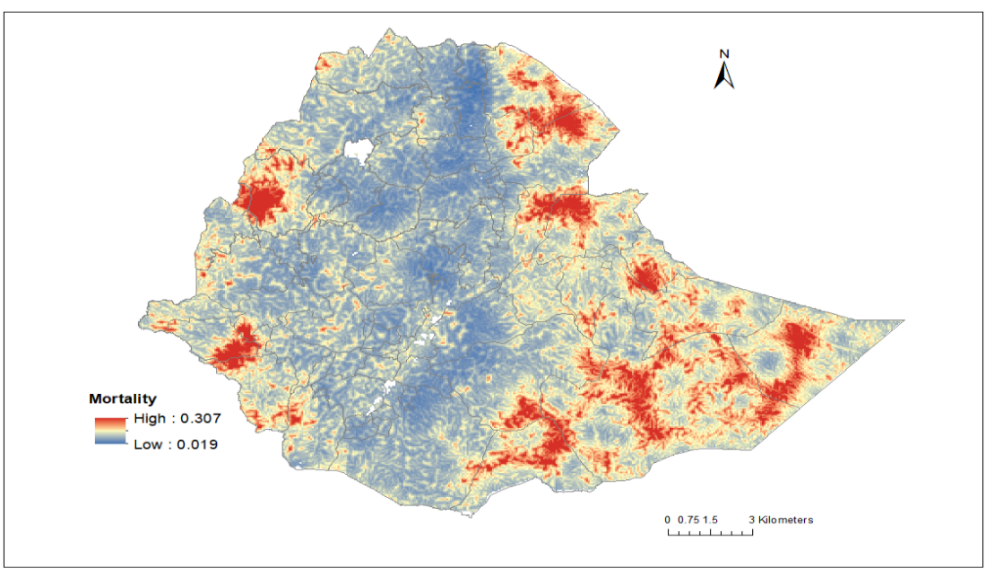
E)

*S2 Fig: The predicted geospatial map for under-five mortality in Ethiopia: 2000(A), 2005(B), 2011(C), 2016(D), and 2019(E).*

Supplement: S2 Fig — (DOCX) [file pgph.0001504.s003.docx]
