# Supplementary material for: Spatiotemporal distributions of under-five mortality in Ethiopia between 2000 and 2019
Source: PLOS Glob Public Health. 2023 Mar 27;3(3):e0001504. doi: 10.1371/journal.pgph.0001504 (PMC10042344; doi:10.1371/journal.pgph.0001504)
Supplement: Attachment — Submitted filename: Point by point response to the reviewers.docx [file pgph.0001504.s004.docx]

06 Dec 2022

Prevost Jantchou
Academic Editor
PLOS Global Public Health

Subject: **Submission of revised manuscript**

We would like to thank for providing us the opportunity to revise our manuscript entitled “Spatiotemporal distributions of under-five mortality in Ethiopia between 2000 and 2019” PGPH-D-22-01440.

We have carefully considered all the comments provided by the editor and reviewer and revised the manuscript accordingly. We believe that the comments have significantly improved the quality of the manuscript.

Additional Editor Comments (if provided):

Thanks for submitting the work to PLOS GLOBAL PUBLIC HEALTH. The reviewers have completed the review of your manuscripts and here are the comments that need to be addressed.

**Response:** We thank both the editor and reviewers for taking their time to review our manuscript. We have addressed all the comments below and revised the manuscript based on the comments

Reviewer 1

1/ This study is very welcome to provide useful data on under 5 mortality in Ethiopia and then focus healt care interventions in appropriated areas.

**Response:** We thank the reviewer for the important comment

2/ It would have been useful to break down the data on mortality according to the age of the children because mortality causes at ages 6 months and 4 years could be very different. Do you have such data?

**Response:** We really appreciate the reviewer for this comment. However, we couldn’t stratify the analysis based on age group suggested by the review as we do not have such data for the denominator.

3/ The main limitation to publishing this paper at this stage is the English. Reviewing the global wording of the manuscript would be mandatory. See below some examples:
line 61-62 : wording is not correct
line 87 : children were participated
Line 111 : was substantially declined

**Response:** We have now thoroughly revised the manuscript. All the language and typos errors are now corrected.

**Reviewer 2**

Congratulations on your paper! It is a fine piece of work and adds relevant information about under-five mortality.

There are some comments to share:
Background: concise, straightfoward, shows essential data.

**Response:** We thank the reviewer for taking the time to provide important comments. we have addressed all the comments below.

Methods:
It is very important to contextualize readers about the country reality.

**Response:** We have now provided additional background information about the under-five mortality for the study area (Ethiopia): “According to the UN Inter-agency Group for Child Mortality Estimation (UNICEF, WHO, World Bank, UN DESA Population Division ) 2021, under-five mortality was 49 deaths per 1000 live birth in Ethiopia.”
Data was obtained from different sources, what can compromise your results. However, the model you have used explains your argument.
**Response:** We have selected potential covariate based on biological plausibility and availability of nationwide data. We have now revised the methods section to address the reviewer comment.

Results:
Great figures! Organized tables.
There a typo mistake at Line 87: probably you mean Table 2, not Table 1, as you mention at the end of the paragraph.

**Response:** We have now corrected the typos, thank you so much.

Discussion:
Well designed, relevant information.
In the Result section, you present data by region (Western, Northeastern, Eastern, and Northcentral). Here you cite specific districts. It is difficult to relate to the figures if there is no mark on the map. By combining both information, citing region and district, readers could better understand you data.
Comparing Map A and Map E on Figure 3, there is a decline on mortality during the years. Did you find any reason for that? It would be very interesting to understand what happened.

**Response:** Thank you for these important comments. As you said there is a decline in mortality as we compare Map A to Map E in figure 3, hence, Map A represents the data from 2000 EDHS and Map E represent the data of 2019 EDHS. The possible reasons were presented in the discussion sections of the manuscript (see Line 117 to 123).

Conclusion:
Concise and clear.

**Response:** We thank you for the reviewer comments.
